# Change in Adverse Events After Enrollment in the National Surgical Quality Improvement Program: A Systematic Review and Meta-Analysis

**DOI:** 10.1371/journal.pone.0146254

**Published:** 2016-01-26

**Authors:** Joshua Montroy, Rodney H. Breau, Sonya Cnossen, Kelsey Witiuk, Andrew Binette, Taylor Ferrier, Luke T. Lavallée, Dean A. Fergusson, David Schramm

**Affiliations:** 1 The Ottawa Hospital Research Institute, Ottawa, Ontario, Canada; 2 School of Medicine, University of Ottawa, Ottawa, Ontario, Canada; 3 Department of Epidemiology and Community Medicine, University of Ottawa, Ottawa, Ontario, Canada; 4 Division of Urology, Department of Surgery, The Ottawa Hospital, Ottawa, Ontario, Canada; 5 Department of Otolaryngology-Head and Neck Surgery, The Ottawa Hospital, Ottawa, Ontario, Canada; Toronto Western Hospital, CANADA

## Abstract

**Background:**

The American College of Surgeons’ National Surgical Quality Improvement Program (NSQIP) is the first nationally validated, risk-adjusted, outcomes-based program to measure and compare the quality of surgical care across North America. Participation in this program may provide an opportunity to reduce the incidence of adverse events related to surgery.

**Study Design:**

A systematic review of the literature was performed. MedLine, EMBASE and PubMed were searched for studies relevant to NSQIP. Patient characteristics, intervention, and primary outcome measures were abstracted. The intervention was participation in NSQIP and monitoring of Individual Site Summary Reports with or without implementation of a quality improvement program. The outcomes of interest were change in peri-operative adverse events and mortality represented by pooled risk ratios (pRR) and 95% confidence intervals (CI).

**Results:**

Eleven articles reporting on 35 health care institutions were included. Nine (82%) of the eleven studies implemented a quality improvement program. Minimal improvements in superficial (pRR 0.81; 95% CI 0.72–0.91), deep (pRR 0.82; 95% CI0.64–1.05) and organ space (pRR 1.15; 95% CI 0.96–1.37) infections were observed at centers that did not institute a quality improvement program. However, centers that reported formal interventions for the prevention and treatment of infections observed substantial improvements (superficial pRR 0.55, 95% CI 0.39–0.77; deep pRR 0.61, 95% CI 0.50–0.75, and organ space pRR 0.60, 95% CI 0.50–0.71). Studies evaluating other adverse events noted decreased incidence following NSQIP participation and implementation of a formal quality improvement program.

**Conclusions:**

These data suggest that NSQIP is effective in reducing surgical morbidity. Improvement in surgical quality appears to be more marked at centers that implemented a formal quality improvement program directed at the reduction of specific morbidities.

## Introduction

The Veterans Affairs Surgical Quality Improvement Program (VASQIP), was a program developed in the 1990s to evaluate risk-adjusted surgical quality [1].The program was successful and eventually expanded to non-VA hospitals. The American College of Surgeons’ National Surgical Quality Improvement Program (NSQIP) evolved from VASQIP in 2005, and is an outcomes-based initiative to measure and compare risk-adjusted adverse events between hospitals [2]. As of 2013, 492 hospitals contributed peri-operative data to NSQIP [2]. NSQIP data are collected by trained Surgical Clinical Reviewers through a standardized and validated process. This process includes specific educational requirements and training, inter-rater reliability checks, and regular audits to ensure accuracy of data entered by Surgical Clinical Reviewers. In 2008, the overall NSQIP surgical data collection agreement rate was 98.4%, indicating highly reliable and accurate information [3].

For each patient captured in NSQIP, up to 135 variables are collected and entered into the database, including demographic data, baseline comorbidities, operative information, and 30-day post-operative adverse events and mortality. While there is some variability in case sampling, most hospitals with greater than 1,680 surgical cases per year enter the first 40 eligible cases in each eight-day period. In contrast, hospitals with less than 1,680 cases per year usually submit data from all surgical cases[4].

The information provided to NSQIP is used to identify differences in the incidence of peri-operative adverse events across specialties and between similar-procedure-volume institutions. Institutions are informed of their performance through Individual Site Summary Reports (ISSR) that are generated semi-annually by NSQIP. NSQIP reports include: total cases, number of adverse events, deaths, expected event rate based on pre-operative risk, logistic model observed/expected ratios with 95% confidence intervals (or hierarchical model odds ratios, as of July 2011), outlier status, and hospital decile rank. Relative deficiencies may be targeted by hospitals for quality improvement and the impact of quality improvement initiatives may be tracked over time using NSQIP reports. Institutions may or may not implement formal quality improvement programs to address sub-standard outcomes[5].

We performed a systematic review and meta-analysis to determine whether participation in NSQIP is associated with a reduction in peri-operative adverse events. We also examined whether simple monitoring of outcomes is sufficient to induce improvements in hospital-specific surgical outcomes, or whether quality improvement initiatives are necessary. We hypothesized that a directed quality improvement program would result in greater adverse event reductions.

## Methods

### Information Source and Search

A comprehensive search of Medline, Embase, and Pub Med was performed (S1 File). Two medical librarians at The Ottawa Hospital assisted in the planning and execution of the search. Since NSQIP is primarily a North American initiative, we searched for English language articles only, published between January 1947 and May 24^th^ 2013 using the search terms NSQIP and National Surgical Quality Improvement Program. All duplicate studies were removed and two reviewers independently screened article titles and abstracts. The reviewers reconciled any disagreements on which full text articles would be obtained for further screening.

### Eligibility criteria & study selection

To be eligible for this review, articles had to report data collected from NSQIP-participating hospitals, and had to present evidence of monitoring of the Individual Site Summary Report or the implementation of a quality improvement program. Included studies compared surgical data before and after commencement of either 1) monitoring the Individual Site Summary Reports (ISSRs) only, or 2) the implementation of a formal quality improvement program in addition to ISSRs. Studies which implemented a formal quality improvement program first identified potential areas for improvement using data provided by their Individual Site Summary Reports. Our primary objective was to determine the degree of surgical quality, defined as the incidence of 30-day morbidities and mortality associated with surgical procedures. We defined formal quality improvement programs as the implementation of protocols or practice guidelines within the hospital with the specific aim of reducing surgical morbidity and/or mortality.

The following categories of articles were excluded from this review:(1) Articles not available in English;(2) Studies that did not compare surgical quality data before and after either the monitoring of the NSIQP Individual Site Summary Reports or the implementation of a quality improvement program;(3) Case studies/case series;(4) Conference abstracts;(5) Commentaries, editorials and review articles;(6) Systematic reviews; and (7) Studies which included results from VASQIP patients. Although systematic reviews were not eligible, they were used to identify additional relevant articles.

### Data collection process & data items of interest

For each article, two reviewers abstracted data using a standardized electronic form. Any disagreements were reconciled by consensus, and if needed, a third party. For studies that reported surgical outcome data over multiple years before and after joining NSQIP, we considered the first year of data presented as pre-quality improvement and the most recent year of data as post-quality improvement implementation. When the number of adverse events was reported as a proportion of the total number of patients, the number of events was calculated by multiplying the total number of patients in the study by the proportion of patients with the adverse event. Publication date, country of study origin, duration of follow-up, and study population size were also collected. Patient characteristics and the type of formal quality improvement program utilized were recorded when available. Outcome data collected included any individually reported adverse event, overall 30-day morbidity, and mortality before and after implementation of the quality improvement initiatives.

### Quality of Studies

There are no validated methods for assessing the quality of observational studies included in a systematic review. We therefore conducted an assessment of study quality based on STROBE guidelines for reporting of observational studies[6].

### Analysis

Overall pooled estimates of the risk ratios (pRR) with 95% confidence interval (CI) for each outcome (e.g. superficial surgical site infections) were calculated using random effects models. In addition, studies were stratified by use of a specific quality intervention or not. No statistical testing was performed to determine the association between quality intervention and adverse events. Funnel plots were generated to assess potential publication bias. All analysis was done using the Comprehensive Meta Analysis Software, version 2.2.064.

## Results

**Fig 1:**
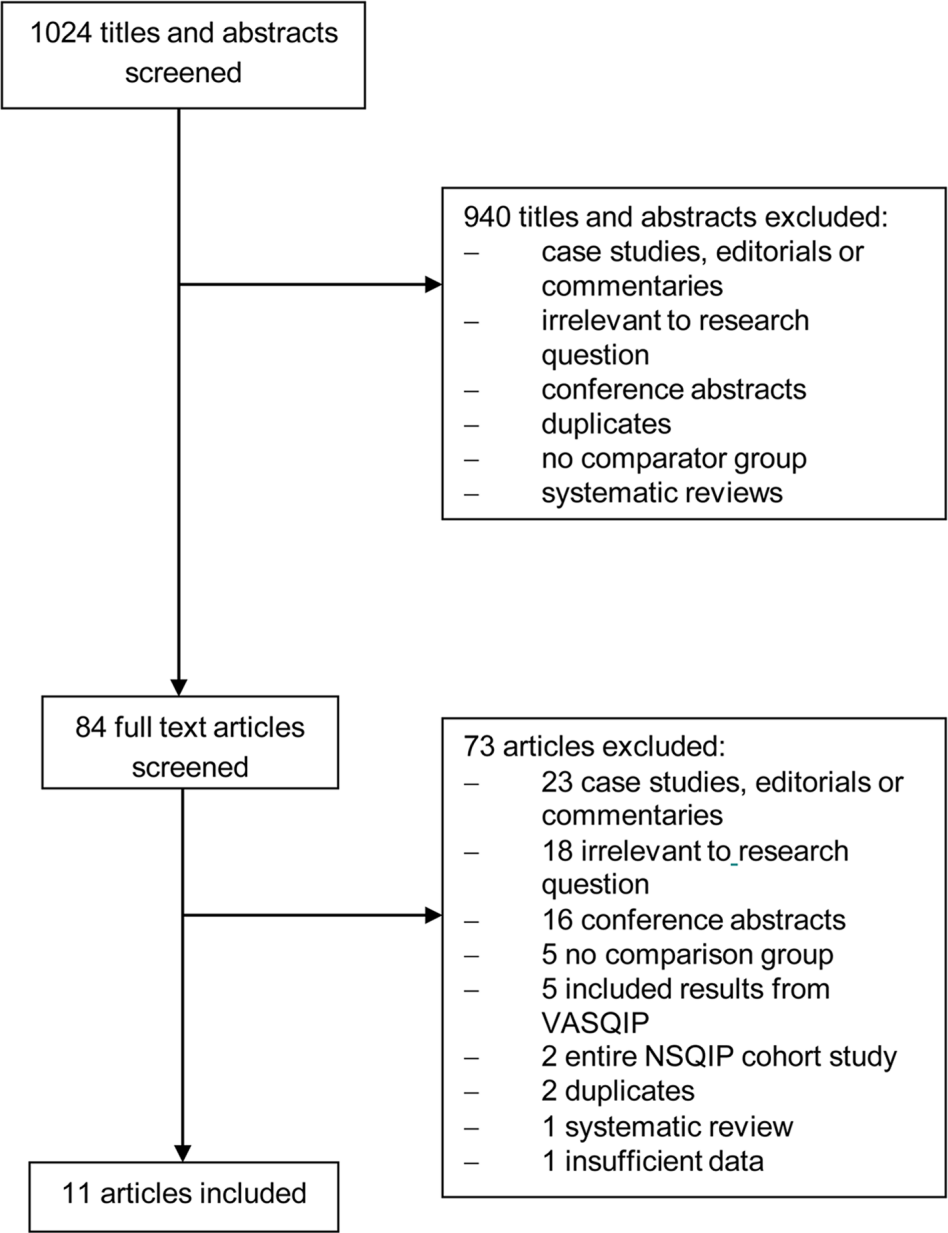
Study screening and selection flow chart. NSQIP = National Surgical Quality Improvement Program; VASQIP = Veteran’s Affairs Surgical Quality Improvement Program.

Of the 1024 titles and abstracts identified by the electronic search, 940 were excluded for being irrelevant or for not meeting our inclusion criteria (Fig 1). A total of 84 full text articles were independently reviewed and 73 were excluded. Eleven full-text articles were abstracted and included in the analysis. Two of the included articles reported data from state-wide cohorts, one within 10 Tennessee hospitals, and one within 16 Michigan hospitals. Therefore, data from 35 institutions, representing 43,010 patients were included in the analysis.

### Characteristics of Included Trials

Included articles were published between January 2010 and January 2013 from hospitals in the United States. Surgical quality data were collected between 2005 and 2011, with specific years varying between studies. Colorectal procedures were the most commonly analyzed surgeries (n = 4), followed by vascular (n = 2), non-specific general surgery (n = 1), otolaryngology (n = 1), non-cardiac (n = 1), intra-abdominal (n = 1) and hepatopancreatobiliary (n = 1) (Table 1).

**Table 1:** Characteristics of included studies.

Author	Data collection period	Institution(s)	N	Primary Outcome(s) measured	Surgery Type	QI (Y/N); Year Initiated	Intervention
Berenguer, 2010^14^	2006–2008	Memorial University Medical Center, Savannah, GA	197	SSI	Colorectal	Y; 2007	SCIP protocol
Bliss, 2012^10^	2010–2011	Saint Francis Hospital and Medical Center, Hartford, CT	319	30-day morbidity	General	Y; 2010	Surgical staff participated in a 3-session team-based training program, followed by implementation of a standardized protocol using pre-op briefing and post-op debriefing checklists
Ceppa, 2013^15^	2007–2009	Indiana University Hospital	895	SSI	Hepatopancreato-biliary	Y; 2008	Standardization of post-opoxygenation, glucose control, and drain and wound management. Careful attention to pre-op nutrition, peri-op antibiotic management, blood transfusions, glycaemic control, temperature control, surgical technique and wound protection
Cima, 2013^16^	2009–2011	Rochester Methodist Hospital	729	SSI	Colorectal	Y; 2010	Surgical care bundle introduced. Categories included; patient cleansing, antibiotic administration, closing protocol at time of fascia closure, patient and hand hygiene, ensure dress removal within 48 hrs, and post-hospitalization processes
Compoginis, 2013^17^	2008–2010	Huntington Hospital, Pasadena, CA	478	SSI	Vascular	Y; 2009	Changing of surgical prep solution and hand washing brushes with chlorhexidine; increase pre-op dose of cefazolin in non-dialysis patients; intra-op redosing of antibiotics in cases > 4 hours; discontinue prophylactic antibiotics within 24 hours of operation; use of supplemental oxygen at an FIO2 of 80% intra-op and in immediate post-op period; routine use of warming devices.
Guillamonde-gui, 2012^9^	2009–2010	Tennessee Surgery Quality Collaborative^a^	29106	Mortality and Post-op complications	General	N	N/A
Henke, 2010^11^	2005–2008	16 Michigan Hospitals	5862	30-day morbidity	Vascular	N	N/A
Lutfiyya, 2012^18^	2006–2011	Kaiser Sunnyside Medical Center, Clackamas, OR,	625	SSI	Colorectal	Y; 2009	Colorectal surgery care bundle: pre-op(SSI education, encourage smoking cessation 30 days before surgery, etc.); Holding (start insulin if blood glucose> 140 mg/gL, remove hair with clippers, apply forced warm air gown); intra-op (antibiotics and prophylactic antimicrobial agents, administer antimicrobial agents on time, etc), post-op (control blood glucose levels, use silver impregnated or polyhexamethylenebiguanide dressing for 5 days, etc.)
Stachler, 2010^12^	2006–2008	Henry Ford Hospital, Detroit, MI	78	DVT	Otolaryngology head and neck surgeries	Y; 2007	Implemented strict protocols and practice plans (flow diagram in paper)
Wick, 2012^19^	2009–2011	John Hopkins Hospital, Baltimore, MD	602	SSI	Colorectal	Y; 2010	CUSP and SCIP guidelines; SSI prevention interventions (standardization of skin prep and pre-op wash cloths, selective elimination of mechanical bowel prep, patient warming pre-anesthesia, enhanced sterile techniques, and addressing lapses in prophylactic antibiotics)
Wren, 2010^13^	2005–2008	VA Palo Alto Health Care System, Palo Alto, CA	3319	Pneumonia	Non cardiac	Y; 2007	Education to nursing staff about pneumonia prevention, cough and deep-breathing exercises with incentive spirometer, oral hygiene with chlorhexidine swabs b.i.d., ambulation with good pain control, head-of-bed elevation to ≥ 30 degrees and sitting up for meals, quarterly discussion of program progress, pneumonia bundle documentation, and computerized physician pneumonia-prevention orders

^a^ Erlanger Hospital (Chattanooga, TN), Vanderbilt University Hospital (Nashville, TN), St Francis Hospital (Memphis, TN), Baptist Memorial Hospital (Memphis, TN), Cookeville Regional Medical Center (Cookeville, TN), Jackson Madison County General Hospital (Jackson, TN), Johnson City Medical Center (Johnson City, TN), Methodist University Hospital (Memphis, TN), Parkwest Medical Center(Knoxville, TN), and the University of Tennessee Medical Center (Knoxville, TN). CUSP = Comprehensive unit-based safety program; FIO2 = Fraction of inspired oxygen; QI = Quality Intervention; SSI = Surgical site infection; SCIP = Surgical care improvement project

Nine (82%) of the 11 studies implemented a quality improvement program. Of these, one study used Surgical Care Improvement Project (SCIP) guidelines[7], while one study used both SCIP and Comprehensive Unit-based Safety Program (CUSP) guidelines[8]. Two studies used modifications of the SCIP guidelines. Five studies created their own formalized quality improvement program that was not based on any specific guidelines. The remaining two studies did not institute a quality improvement program; however, all 11 studies used NSQIP performance feedback from ISSRs to monitor their outcomes both before and after the implementation of NSQIP (Table 1).Since studies which implemented a formal quality improvement program had first monitored their results by using their ISSR’s, any changes in morbidity or mortality rates were in addition to the changes seen via the monitoring of ISSR’s alone.

The quality of included studies was evaluated based on an adaptation of STROBE guidelines. All included studies reported on study design, study setting, variables analyzed, statistical methods, outcome data, and summary/interpretation of key findings. Overall, each included study contained at least 60% of the recommended STROBE checklist items (S1 Table).

### Overall 30-day post-operative mortality

Only one study presented data on 30-day post-operative mortality[9]. In this study of 10 Tennessee hospitals that did not implement a specific initiative for mortality reduction, no decrease in mortality was observed after joining NSQIP (RR 1.03; 95%CI 0.88–1.19).

### Overall 30-day post-operative morbidity

Two studies presented data on 30-day post-operative morbidity (defined as any complication within 30 days of surgery)[10,11]. In one of these studies, no specific quality improvement program was used and a small reduction in morbidity was observed (RR 0.87; 95%CI0.77–0.99)[11]. In the other study, a quality improvement program was instituted and consisted of a three-session team-based training program for surgical staff, followed by the implementation of a standardized protocol using preoperative briefing and post-operative debriefing checklists[10]. The reduction in morbidity was greater at that institution, but it was not statistically significant (RR 0.52; 95% CI 0.23–1.18).

### Surgical infections

**Fig 2:**
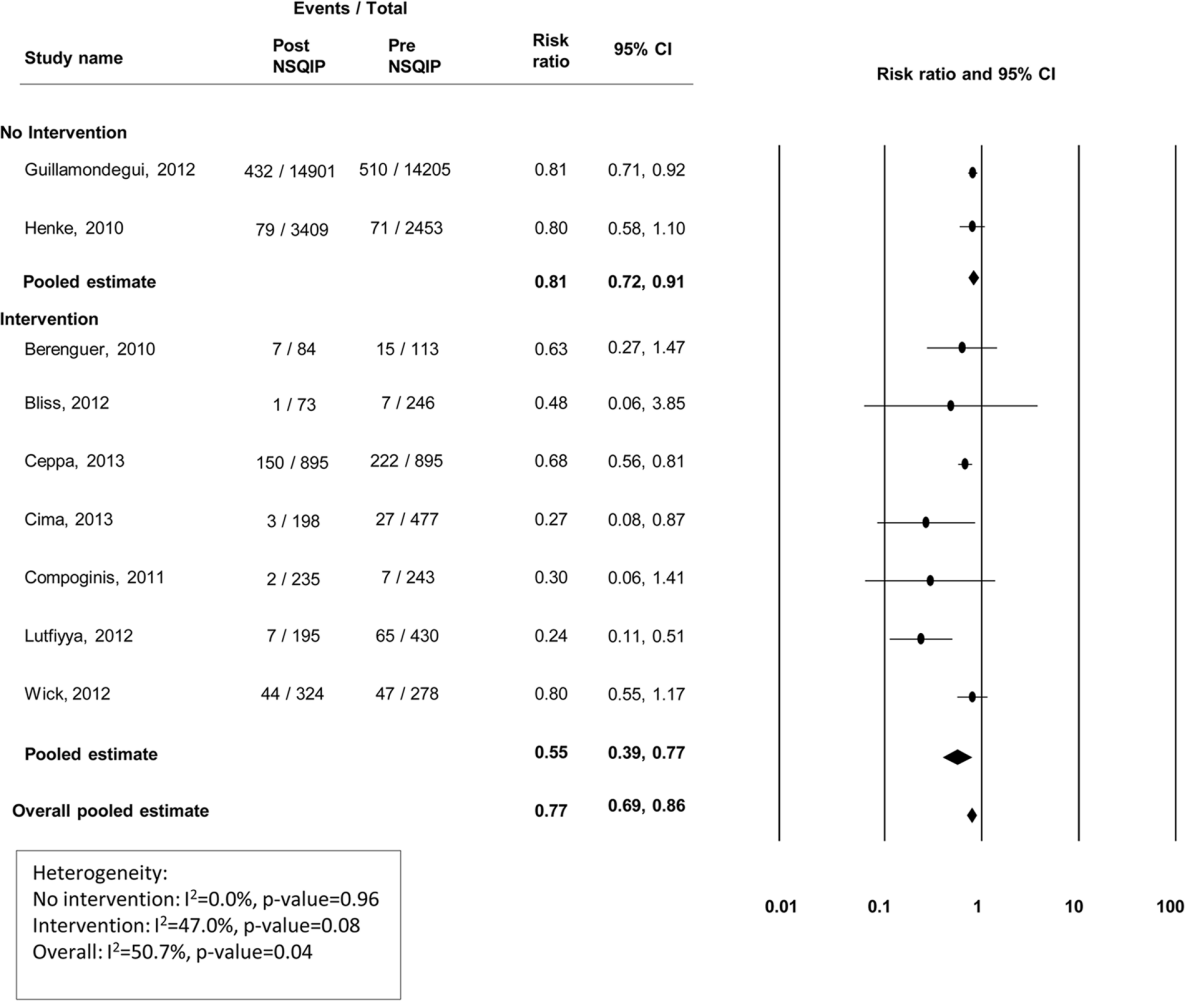
Risk ratios (95% CI) and pooled estimates for superficial surgical site infections, pre vs. post-NSQIP implementation, stratified by intervention or no intervention to reduce infection.

**Fig 3:**
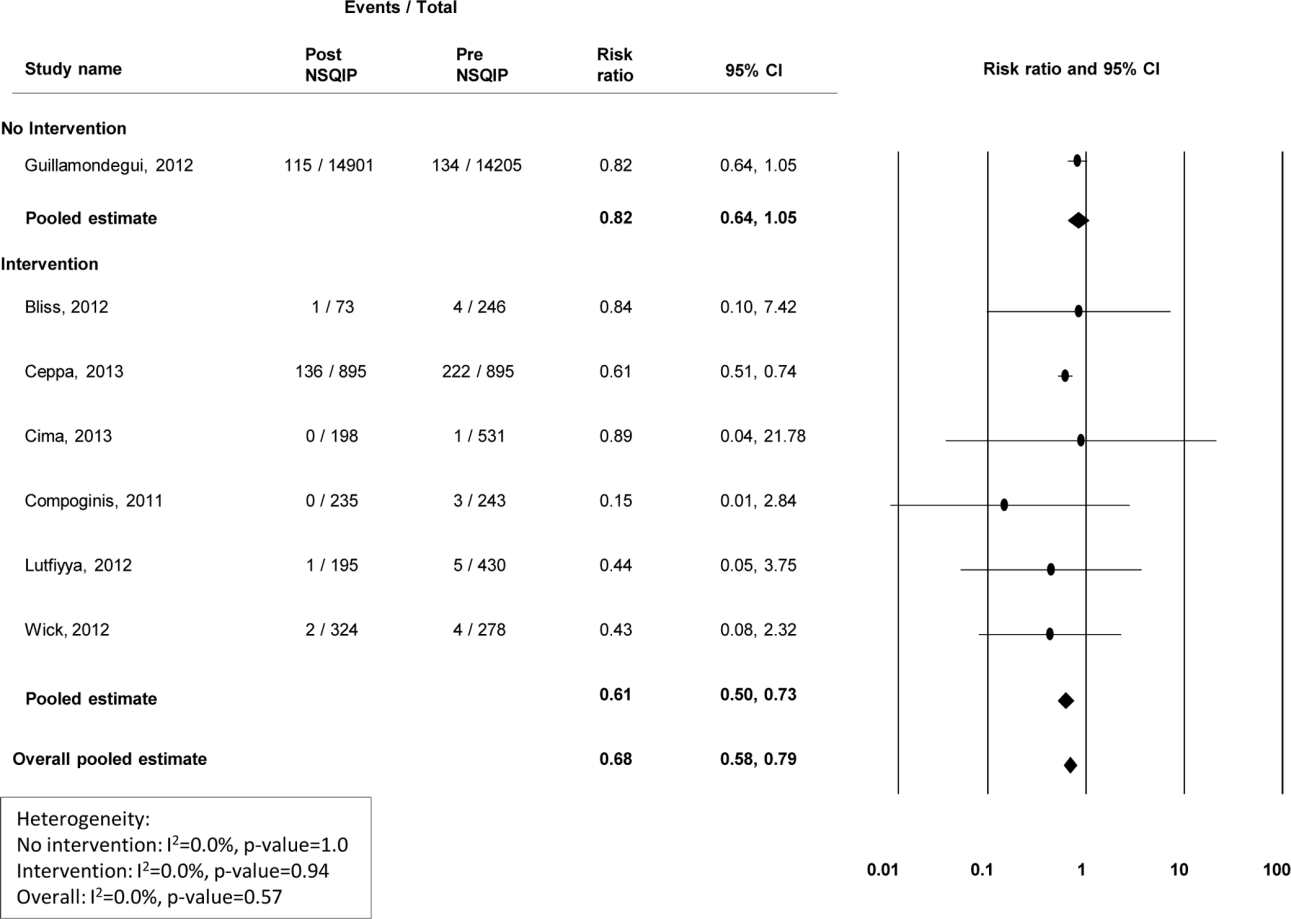
Risk ratios (95% CI) and pooled estimates for deep surgical site infections pre vs. post-NSQIP implementation, stratified by intervention or no intervention to reduce infection.

**Fig 4:**
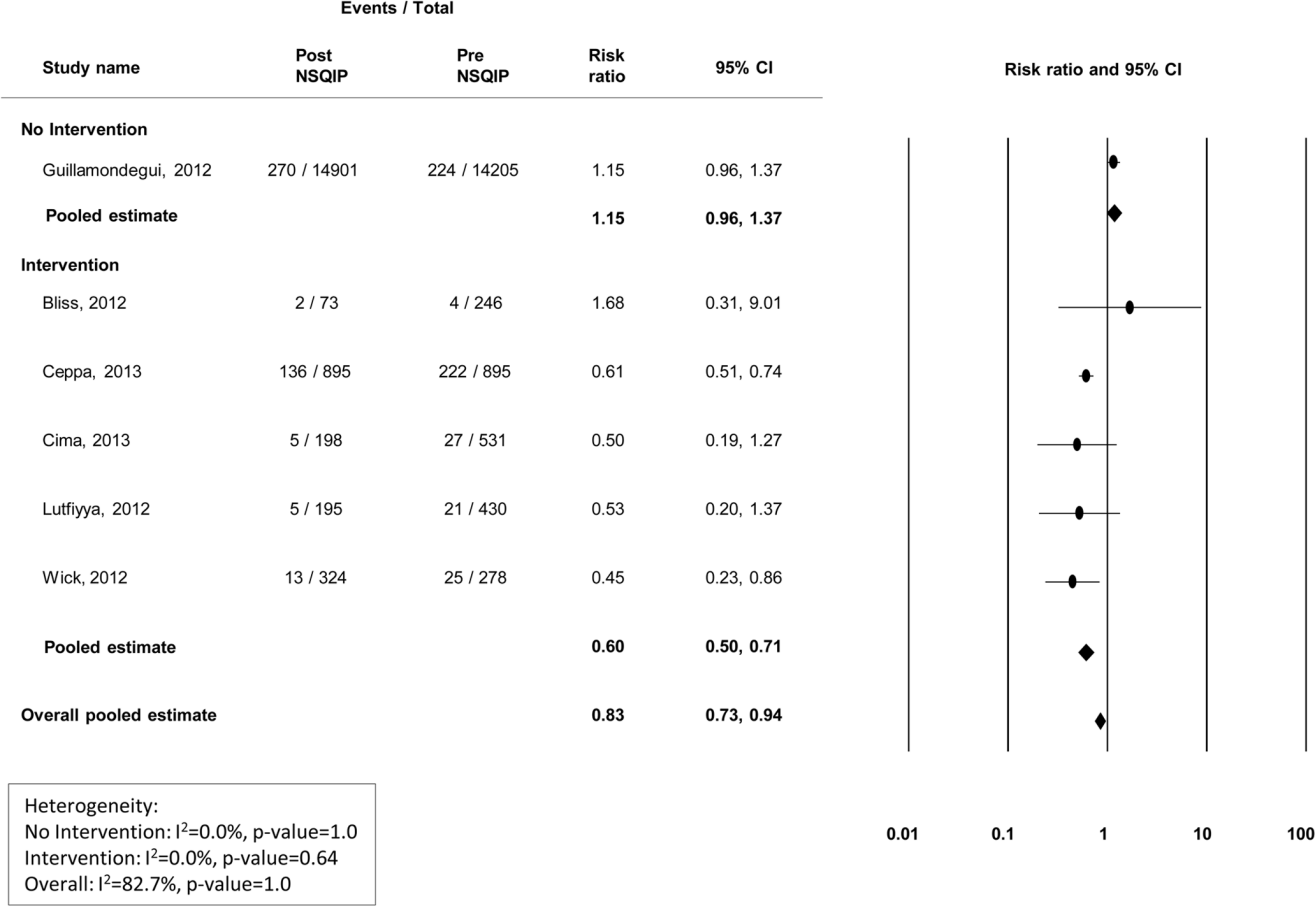
Risk ratios (95% CI) and pooled estimates for organ/abdominal space infections pre vs. post-NSQIP implementation, stratified by intervention or no intervention to reduce infection.

**Fig 5:**
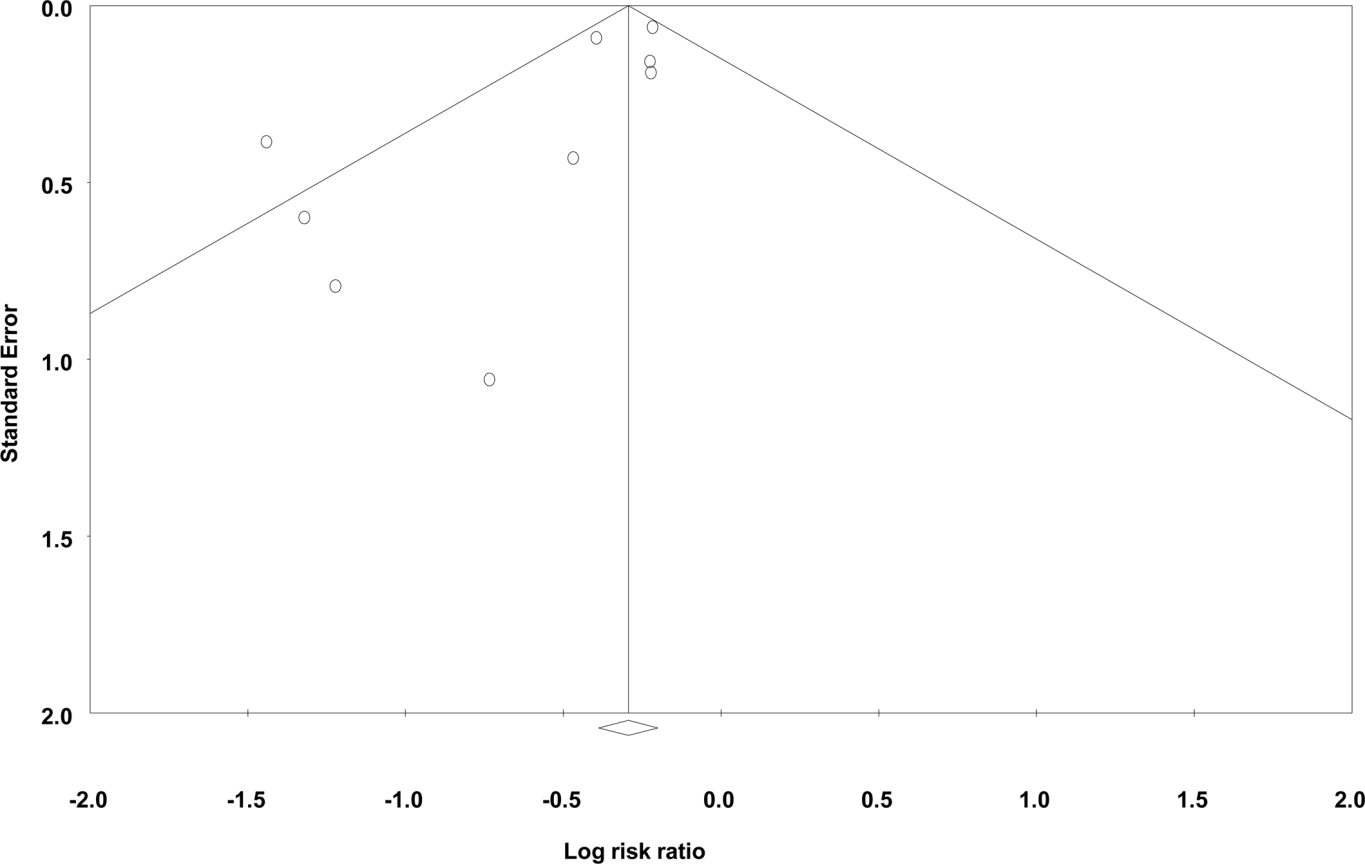
Funnel plot of standard error vs. log risk ratio for superficial surgical site infections.

**Fig 6:**
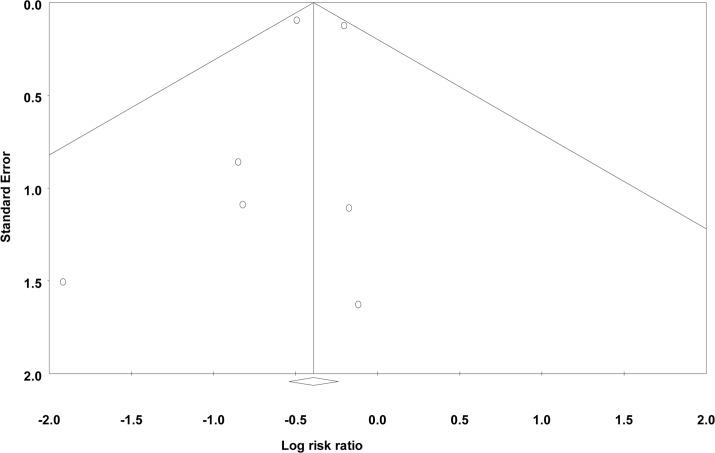
Funnel plot of standard error vs. log risk ratio for (b) deep surgical site infections.

**Fig 7:**
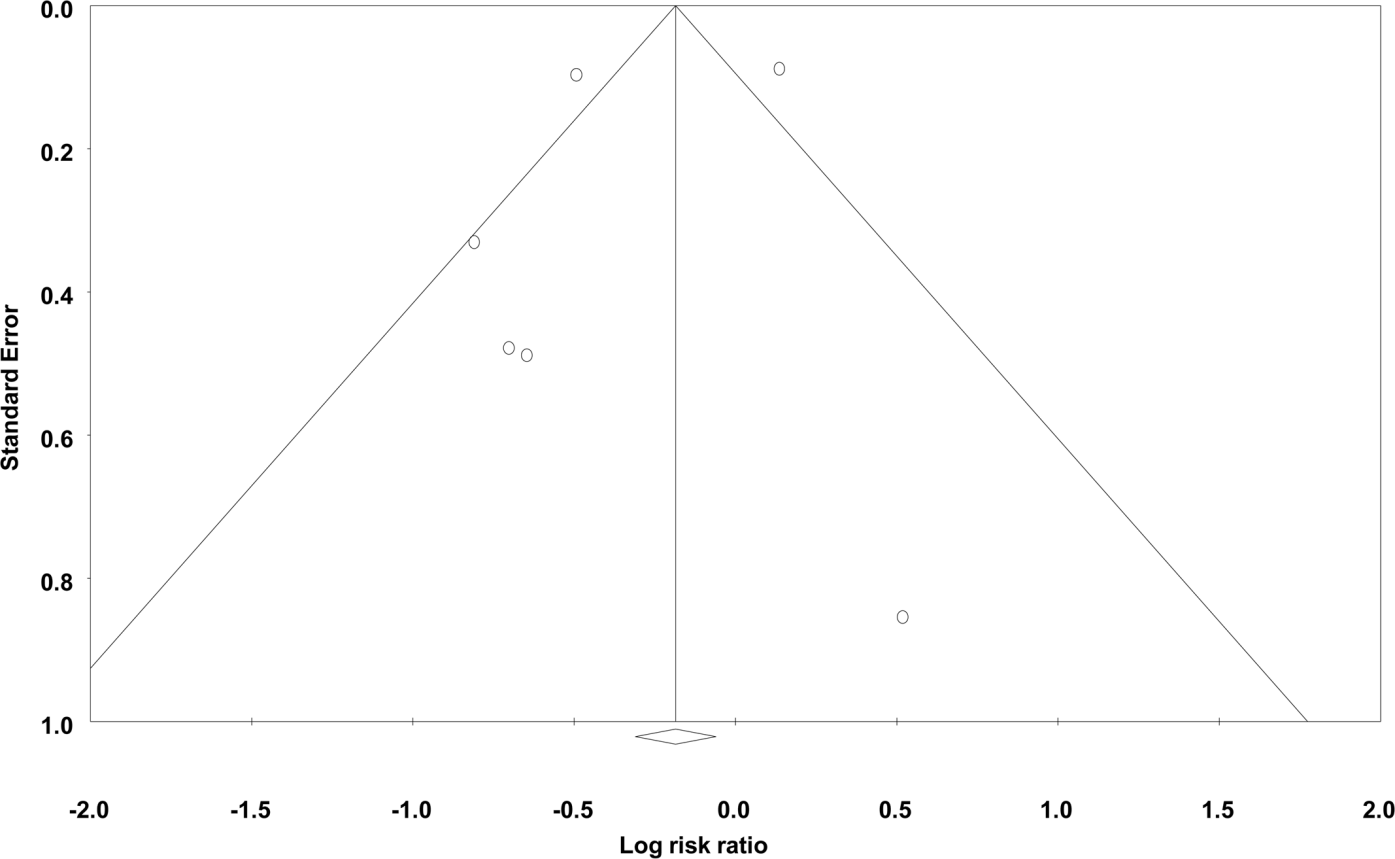
Funnel plot of standard error vs. log risk ratio for organ/abdominal space infections.

Nine studies presented data on the incidence of superficial surgical site infections (SSIs), 7 presented deep SSI information, and 6 presented organ/abdominal space infection information (Figs 2–4). Modest improvements in superficial (Fig 2) and deep wound infections (Fig 3) were observed at centers that did not institute a quality improvement program, with pooled risk ratios of 0.81(95% CI 0.72–0.91) and 0.82 (95% CI 0.64–1.05), respectively. However, no improvement was observed for organ/abdominal space infections, with a pooled risk ratio of 1.15 (95% CI 0.96–1.37) (Fig 4). Among centers that reported specific interventions for the prevention/treatment of SSIs, reductions in infection rates were substantial. For superficial, deep, and organ/abdominal space SSIs, the pooled risk ratios were 0.55 (95% CI 0.39–0.77), 0.61 (95% CI 0.50–0.73) and 0.60 (95% CI 0.50–0.71), respectively (Figs 2–4). Funnel plots to assess potential selection bias are presented in Figs 5–7.

### Thromboembolic and other complications

Two studies reported on deep venous thrombosis complications, one which implemented a quality improvement program, and one did not. The study which did not implement a quality improvement program[9]observed an increase in the rate of deep venous thrombosis, with a relative risk of 1.35(95% CI1.04–1.76). The study which did implement a quality improvement program [12] observed a decrease in the incidence of deep venous thrombosis (RR 0.3; 95%CI 0.002–5.42). For all other complications, such as pneumonia [13] and septic shock, modest or no improvement was observed unless a quality improvement initiative was used (S2 Table).

## Discussion

The American College of Surgeons’ National Surgical Quality Improvement Program provides comprehensive and accurate performance feedback to hospitals. However, upon conducting a systematic review of the literature published from NSQIP-participating hospitals, our analysis suggests that implementing a formal quality improvement program may be more effective than merely monitoring surgical outcomes using NSQIP Individual Site Summary Reports. Indeed, in NSQIP hospitals that implemented a quality improvement program, there was a reduction in the incidence of almost all post-operative complications.

This review captured studies implementing a broad range of quality programs. All of the seven studies that reported such initiatives for a common post-operative complication of wound infection reported a successful reduction in surgical site infections[10, 14–19]. Common components of these programs were: warming of the patient prior to surgery/maintaining peri-operative and post-operative normothermia, proper pre-operative, peri-operative, and post-operative use of antibiotics, and peri-operative and post-operative glucose control. An eight-step program to reduce pneumonia rates was introduced in one study that included education of all surgical ward nursing staff on their role in pneumonia prevention and twice daily oral hygiene with chlorhexidine swabs. At that center, pneumonia relative risk was reduced from 0.8% to 0.2%. Unplanned intubation rates were successfully reduced by implementing a three-step program, including the creation of a working group, the identification of operational problems, and development of processes to change practice[10]. Overall 30-day morbidity was reduced by having surgical staff participate in a three session team-based training program followed by the implementation of a standardized protocol using preoperative briefing and post-operative debriefing check lists[10]. In summary, the quality improvement programs were often directed at specific outcomes and, in general, were highly effective. Although shown to be effective in reducing specific morbidities, the cost of participating in the NSQIP program must be taken into account, especially for small-volume institutions.

There are several potential limitations to this comprehensive review. As with all systematic reviews, we are limited by the quality of the published studies and authors may have preferentially reported programs that have shown an improvement, resulting in a publication bias (Figs 5–7). It is possible that improvements seen in the incidence of SSIs after a formal quality improvement program were due to publication bias. We were not able to adjust for differences in case-mix when comparing evaluation periods. Therefore, the true impact of NSQIP participation may be over or under-estimated if the baseline patient risk changed over time at individual institutions. A limitation of the present analysis is the fact that the majority of NSQIP participating institutions are located in English speaking countries and that all publications were from the United States. A main limitation of our review was the small number of studies included in the final analysis. In certain instances, outcomes such as any 30-day morbidity or sepsis were only reported by two studies and overall 30-day mortality was only reported by one study. Therefore, our ability to form definitive conclusions about these outcomes is limited. Total morbidity rates were only discussed by two of the included papers. While improvements in the incidence of specific morbidities were reported by other papers, conclusions regarding the true, overall surgical quality improvement were limited by the small number of publications regarding overall morbidity.

Most hospitals within the United States are accredited by the Joint Commission, which requires an intrinsic quality improvement process. This means that the majority of the hospitals in our study had an ongoing improvement program in place prior to joining NSQIP. However, the intrinsic program required by the Joint Commission targets overall patient care improvement within a hospital. There is no identification of potential areas for improvement or areas of weakness at each specific hospital. Quality improvement programs directed at reducing the incidence of specific morbidities or reducing morbidities associated with a specific procedure are likely results of the NSQIP ISSR’s. Lastly, the phenomenon of regression to the mean may explain some of the improvements observed in hospitals that reported on outcomes that had historically performed worse in than similar-volume institutions. However, this effect on NSQIP institutions appears to be minimal, as NSQIP-related improvements were found to be independent from this effect[5].

## Conclusion

To our knowledge, this is the first systematic review to attempt to evaluate the effectiveness of the National Surgical Quality Improvement Program in improving post-operative morbidity and mortality. No analysis was performed on mortality rates, as there was insufficient data. Our analysis of morbidity rates suggest that implementation of a formal quality improvement program based on NSQIP feedback was more effective compared to passive monitoring of surgical outcomes generated by NSQIP’s Individual Site Summary reports. These findings have noteworthy implications for hospitals that participate in NSQIP but have not yet adopted quality improvement programs to address deficiencies. Establishing and implementing outcome-directed quality improvement programs may be of significant benefit to NSQIP-participating hospitals that aim to improve surgical quality by reducing the incidence of specific post-surgical complications, however it is not known if this translates to overall surgical quality improvement.

## Supporting Information

S1 PRISMA Checklist PRISMA Checklist. (DOCX)

S1 File Search Strategies. (DOCX)

S1 Table Assessment of study quality based on STROBE guidelines. (DOCX)

S2 Table Specific morbidity rates before and after National Surgical Quality Improvement Program implementation. (DOCX)
