# Supplementary material for: Change in Adverse Events After Enrollment in the National Surgical Quality Improvement Program: A Systematic Review and Meta-Analysis
Source: PLoS One. 2016 Jan 26;11(1):e0146254. doi: 10.1371/journal.pone.0146254 (PMC4727780; doi:10.1371/journal.pone.0146254)
Supplement: S1 File — (DOCX) [file pone.0146254.s002.docx]

# S1 File: Search Strategies

Database: EmbaseClassic+Embase<1947 to 2013 May 24>, Ovid MEDLINE(R) In-Process & Other Non-Indexed Citations and Ovid MEDLINE(R) <1946 to Present>

Search Strategy:

--------------------------------------------------------------------------------

1 National Surgical Quality Improvement Program$.tw. (1310)

2 NSQIP$.tw. (961)

3 1 or 2 (1599)

4 remove duplicates from 3 (975)

5 limit 4 to english language (974)

| Recent queries in pubmed | |  |
| --- | --- | --- |
| Search | Query | Items found |
| #10 | Search (#9) AND #4 | 56 |
| #9 | Search (#8) OR #7 | 661 |
| #8 | Search "nsqip" | 349 |
| #7 | Search "national surgical quality improvement program" | 579 |
| #4 | Search publisher [sb] | 429796 |
