# Supplementary material for: Change in Adverse Events After Enrollment in the National Surgical Quality Improvement Program: A Systematic Review and Meta-Analysis
Source: PLoS One. 2016 Jan 26;11(1):e0146254. doi: 10.1371/journal.pone.0146254 (PMC4727780; doi:10.1371/journal.pone.0146254)
Supplement: S1 Table — (DOCX) [file pone.0146254.s003.docx]

**S1 Table: Assessment of study quality based on STROBE guidelines**

| **STROBE Guideline** | **Berenguer,**  **2013** | **Bliss,**  **2012** | **Ceppa,**  **2013** | **Cima,**  **2013** | **Compoginis,**  **2013** | **Guillamondegui,**  **2012** | **Henke,**  **2010** | **Lutfiyya, 2012** | **Stachler,**  **2010** | **Wick,**  **2012** | **Wren,**  **2010** |
| --- | --- | --- | --- | --- | --- | --- | --- | --- | --- | --- | --- |
| **Title and abstract** |  |  |  |  |  |  |  |  |  |  |  |
| 1a. Indicate study design | ✓ | — | — | ✓ | ✓ | — | ✓ | ✓ | ✓ | — | ✓ |
| 1b. Summary of study | ✓ | ✓ | ✓ | ✓ | ✓ | ✓ | ✓ | ✓ | ✓ | ✓ | ✓ |
| **Introduction** |  |  |  |  |  |  |  |  |  |  |  |
| 2. Background/rationale | ✓ | ✓ | ✓ | ✓ | ✓ | ✓ | ✓ | ✓ | ✓ | ✓ | ✓ |
| 3. Objectives | ✓ | — | ✓ | ✓ | ✓ | ✓ | ✓ | ✓ | ✓ | ✓ | ✓ |
| **Methods** |  |  |  |  |  |  |  |  |  |  |  |
| 4. Study design | ✓ | ✓ | ✓ | ✓ | ✓ | ✓ | ✓ | ✓ | ✓ | ✓ | ✓ |
| 5. Setting | ✓ | ✓ | ✓ | ✓ | ✓ | ✓ | ✓ | ✓ | ✓ | ✓ | ✓ |
| 6a. Eligibility criteria | ✓ | ✓ | ✓ | ✓ | ✓ | — | ✓ | ✓ | — | ✓ | ✓ |
| 6b. Matching criteria | NA | NA | NA | NA | NA | NA | NA | NA | NA | NA | NA |
| 7. Variables | ✓ | ✓ | ✓ | ✓ | ✓ | ✓ | ✓ | ✓ | ✓ | ✓ | ✓ |
| 8. Data sources/ measurement | ✓ | ✓ | ✓ | ✓ | ✓ | ✓ | ✓ | ✓ | ✓ | ✓ | ✓ |
| 9. Bias | — | ✓ | — | — | — | — | ✓ | — | — | — |  |
| 10. Study size | NA | NA | NA | NA | NA | NA | NA | NA | NA | NA | NA |
| 11. Quantitative variables | ✓ | ✓ | ✓ | ✓ | ✓ | ✓ | ✓ | ✓ | ✓ | ✓ | ✓ |
| 12a. Statistical methods | ✓ | ✓ | ✓ | ✓ | ✓ | ✓ | ✓ | ✓ | ✓ | ✓ | ✓ |
| 12b. Subgroups and interactions | — | ✓ | — | ✓ | — | — | — | ✓ | — | — | — |
| 12c. Missing data | — | — | — | — | — | ✓ | — | — | — | — | — |
| 12d. Loss to follow-up/matching/sampling | NA | NA | NA | ✓ | NA | NA | NA | NA | NA | ✓ | NA |
| 12e. Sensitivity analyses | — | — | — | — | — | ✓ | — | — | — | — | — |
| **Results** |  |  |  |  |  |  |  |  |  |  |  |
| 13a. Number of participants | ✓ | ✓ | ✓ | ✓ | ✓ | ✓ | ✓ | ✓ | ✓ | ✓ | ✓ |
| 13b. Non-participation | NA | NA | NA | NA | NA | NA | NA | NA | NA | NA | NA |
| 13c. Flow diagram | NA | NA | NA | NA | NA | NA | NA | NA | NA | NA | NA |
| 14a. Participant characteristics | — | ✓ | — | ✓ | ✓ | ✓ | ✓ | — | NA | ✓ | — |
| 14b. Missing data | — | — | — | — | — | NA | — | — | NA | — | — |
| 14c. Follow-up time | NA | NA | NA | NA | NA | NA | NA | NA | NA | NA | NA |
| 15. Outcome data | ✓ | ✓ | ✓ | ✓ | ✓ | ✓ | ✓ | ✓ | ✓ | ✓ | ✓ |
| 16a. Unadjusted/ adjusted estimates | ✓ | ✓ | ✓ | ✓ | ✓ | ✓ | ✓ | ✓ | ✓ | ✓ | ✓ |
| 16b. Category boundaries | NA | NA | NA | ✓ | NA | NA | NA | NA | NA | NA | NA |
| 16c. Relative risk to absolute risk | NA | NA | NA | NA | NA | — | NA | NA | NA | NA | NA |
| 17. Other analyses | NA | ✓ | NA | ✓ | ✓ | ✓ | ✓ | ✓ | — | NA | NA |
| **Discussion** |  |  |  |  |  |  |  |  |  |  |  |
| 18. Key results | ✓ | ✓ | ✓ | ✓ | ✓ | ✓ | ✓ | ✓ | ✓ | ✓ | ✓ |
| 19. Limitations | — | ✓ | ✓ | ✓ | — | ✓ | ✓ | ✓ | — | ✓ | — |
| 20. Interpretation | ✓ | ✓ | ✓ | ✓ | ✓ | ✓ | ✓ | ✓ | ✓ | ✓ | ✓ |
| 21. Generalizability | — | — | — | ✓ | — | — | ✓ | — | — | ✓ | — |
| **Other information** |  |  |  |  |  |  |  |  |  |  |  |
| 22. Funding | — | — | — | — | — | — | — | — | ✓ | — | — |
