# Supplementary material for: Change in Adverse Events After Enrollment in the National Surgical Quality Improvement Program: A Systematic Review and Meta-Analysis
Source: PLoS One. 2016 Jan 26;11(1):e0146254. doi: 10.1371/journal.pone.0146254 (PMC4727780; doi:10.1371/journal.pone.0146254)
Supplement: S2 Table — (DOCX) [file pone.0146254.s004.docx]

S2 Table: Specific morbidity rates before and after National Surgical Quality Improvement Program implementation

| **Study** | **Outcome** | **Quality Intervention (Y/N)** | **Pre-NSQIP**  **#events/Total** | **Post-NSQIP**  **#events/Total** | **Relative Risk (95% CI)** | **Absolute Risk Difference (95% CI)** |
| --- | --- | --- | --- | --- | --- | --- |
| Guillamondegui, 2012^9^ | Deep venous thrombosis | N | 94 / 14205 | 133 / 14901 | 1.35 (1.04,1.76) | 0.002 (0.0003,0.004) |
| Bliss, 2012^11^ | Deep venous thrombosis and pulmonary embolism | Y | 5 / 246 | 0 / 73 | 0.3 (0.002, 5.42) | -0.02 (-0.05,0.006) |
| Guillamondegui, 2012^9^ | Pulmonary embolism | N | 48 / 14205 | 60 / 14901 | 1.19 (0.82,1.74) | 0.0006 (-0.0007,0.002) |
| Guillamondegui, 2012^9^ | Pneumonia | N | 320 / 14205 | 413 / 14901 | 1.23 (1.07,1.42) | 0.005 (0.001,0.009) |
| Henke, 2010^10^ | Pneumonia | N | 76 / 2453 | 72 / 3409 | 0.68 (0.5,0.94) | -0.01 (-0.02,-0.002) |
| Bliss, 2012^11^ | Pneumonia | Y | 7 / 246 | 0 / 73 | 0.22 (0.01,3.85) | -0.03 (-0.06,0.0001) |
| Fuchshuber, 2012^2^ | Pneumonia | Y | 15 / 1500 | 0 / 1600 | 0.03 (0.002,0.51) | -0.01 (-0.02,-0.005) |
| Wren, 2010^19^ | Pneumonia | Y | 13 / 1668 | 3 / 1651 | 0.23 (0.07,0.82) | -0.006 (-0.01,-0.001) |
| Guillamondegui, 2012^9^ | Unplanned intubation | N | 259 / 14205 | 247 / 14901 | 0.91 (0.77,1.08) | -0.002 (-0.005,0.001) |
| Henke, 2010^10^ | Unplanned intubation | N | 69 / 2453 | 75 / 3409 | 0.78 (0.57,1.08) | -0.006 (-0.01,0.002) |
| Guillamondegui, 2012^9^ | Wound disruption | N | 129 / 14205 | 89 / 14901 | 0.66 (0.5,0.86) | -0.003 (-0.005,-0.001) |
| **Study** | **Outcome** | **Quality Intervention (Y/N)** | **Pre-NSQIP**  **#events/Total** | **Post-NSQIP**  **#events/Total** | **Relative Risk (95% CI)** | **Absolute Risk Difference (95% CI)** |
| Bliss, 2012^11^ | Wound disruption | Y | 3 / 246 | 0 / 73 | 0.48 (0.03,9.13) | -0.01 (-0.04,0.01) |
| Guillamondegui, 2012^9^ | Acute renal failure | N | 107 / 14205 | 83 / 14901 | 0.74 (0.56,0.98) | -0.002 (-0.004,-0.0001) |
| Guillamondegui, 2012^9^ | Urinary tract infection | N | 234 / 14205 | 349 / 14901 | 1.42 (1.21,1.68) | 0.007 (0.004, 0.01) |
| Bliss, 2012^11^ | Urinary tract infection | Y | 7 / 246 | 2 / 73 | 0.96 (0.2,4.53) | -0.001 (-0.04,0.04) |
| Guillamondegui, 2012^9^ | Progressive renal insufficiency | N | 75 / 14205 | 82 / 14901 | 1.04 (0.76,1.42) | 0.0002 (-0.002,0.002) |
| Guillamondegui, 2012^9^ | Systemic sepsis | N | 308 / 14205 | 289 / 14901 | 0.89 (0.76,1.05) | -0.002 (-0.006,0.001) |
| Henke, 2010^10^ | Systemic sepsis | N | 76 / 2453 | 61 / 3409 | 0.58 (0.41,0.81) | -0.01 (-0.02, -0.005) |
| Cima, 2013^16^ | Systemic sepsis | Y | 10 / 531 | 6 / 198 | 1.61 (0.59,4.37) | 0.01 (-0.02,0.04) |
| Bliss, 2012^11^ | Systemic sepsis | Y | 10 / 246 | 5 / 195 | 0.63 (0.22, 1.82) | -0.02 (-0.05,0.02) |
| Ozhathil, 2011^21^ | Septic shock | N | 93 / 2629 | 757 / 20882 | 1.03 (0.83,1.27) | 0.001 (-0.007,0.008) |
| Henke, 2010^10^ | Septic shock | N | 61 / 2453 | 44 / 3409 | 0.52 (0.35,0.76) | -0.01 (-0.02, -0.005) |
| Bliss, 2012^11^ | On ventilator >48 hours | Y | 8 / 246 | 0 / 73 | 0.2 (0.01,3.36) | -0.03 (-0.06,-0.003) |
| Fuchshuber, 2012^2^ | On ventilator >48 hours | Y | 38 / 1500 | 20 / 1600 | 0.49 (0.29,0.84) | -0.01 (-0.02,-0.003) |
| **Study** | **Outcome** | **Quality Intervention (Y/N)** | **Pre-NSQIP**  **#events/Total** | **Post-NSQIP**  **#events/Total** | **Relative Risk (95% CI)** | **Absolute Risk Difference (95% CI)** |
| Guillamondegui, 2012^9^ | Stroke/CVA | N | 40 / 14205 | 40 / 14901 | 0.95 (0.62,1.48) | -0.0001 (-0.001,0.001) |
| Guillamondegui, 2012^9^ | Cardiac arrest requiring CPR | N | 80 / 14205 | 77 / 14901 | 0.92 (0.67,1.25) | -0.0005 (-0.002,0.001) |
| Henke, 2010^10^ | Cardiac arrest requiring CPR | N | 29 / 2453 | 27 / 3409 | 0.67 (0.4,1.13) | -0.004 (-0.009,0.001) |
| Guillamondegui, 2012^9^ | Other cardiac occurrences | N | 88 / 14205 | 88 / 14901 | 0.95 (0.71,1.28) | -0.0003 (-0.0021,0.0015) |
